# Resting-state gamma oscillations in adult Autism spectrum disorder: A High-Density EEG study

**DOI:** 10.1192/j.eurpsy.2023.1275

**Published:** 2023-07-19

**Authors:** B. Kakuszi, B. Szuromi, M. Tóth, I. Bitter, P. Czobor

**Affiliations:** 1 Psychiatry and Psychotherapy, Semmelweis University; 2 OMIII Nyírő Gyula Hospital, Budapest, Hungary

## Abstract

**Introduction:**

Autism is neurodevelopmental disorder with a heterogeneous presentation of symptoms, which include disturbances in sensory, motor and cognitive processes, among which social cognitive impairments and social interaction difficulties play prominent role. Despite the fact that these impairments can lead to lifelong disability and difficulties in everyday functioning, their neurobiological basis remains largely unknown. Neural oscillations in the gamma band have been shown to be an important candidate neurobiological marker of higher order cognitive processes and social interactions. Yet, alterations of gamma oscillations in ASD have received little attention in the literature.

**Objectives:**

The aim of the current study was to investigate resting state gamma oscillations in the EEG in order to delineate alterations in ASD as compared to typically developing (TD) subjects in the intrinsic activity of the neural networks that have been linked to social cognitive functioning.

**Methods:**

Resting-state EEGs were obtained in an ongoing study investigating ASD (N=19) and TD subjects (N=15), based on eyes closed condition. EEGs were recorded using a 128-channel BioSemi system. EEG absolute power was investigated in the gamma 30-48Hz frequency band.

**Results:**

Gamma activity was significantly (p<0.05) diminished in multiple brain regions in ASD as compared TD subjects. The diminished gamma activity had a distinctive topographical distribution, which included the left and right inferior temporal gyrus, the right superior temporal gyrus, the TPJ and the right extrastriate areas. Additionally, we found a hemispheric asymmetry in the occipital brain areas with a decrease of gamma activity on the right and an increase in the left hemisphere as compared to TD.

**Conclusions:**

Diminished gamma activity in the above brain areas may represent a cortical dysfunction which can be present due to a reduced capacity to process socially relevant information and a decreased capacity to omit irrelevant stimuli.

Funding: Hungarian Brain Research program,#NAP2022-I-4/2022

**Disclosure of Interest:**

None Declared

